# The Impact of Visualizing Nested Sets. An Empirical Study on Tree Diagrams and Unit Squares

**DOI:** 10.3389/fpsyg.2016.02026

**Published:** 2017-01-06

**Authors:** Katharina Böcherer-Linder, Andreas Eichler

**Affiliations:** ^1^Institute of Mathematics Education, University of Education, FreiburgFreiburg, Germany; ^2^Institute of Mathematics, University of KasselKassel, Germany

**Keywords:** Bayesian reasoning, visualization, unit square, tree diagram, nested sets

## Abstract

It is an ongoing debate, what properties of visualizations increase people’s performance when solving Bayesian reasoning tasks. In the discussion of the properties of two visualizations, i.e., the tree diagram and the unit square, we emphasize how both visualizations make relevant subset relations transparent. Actually, the unit square with natural frequencies reveals the subset relation that is essential for the Bayes’ rule in a numerical and geometrical way whereas the tree diagram with natural frequencies does it only in a numerical way. Accordingly, in a first experiment with 148 university students, the unit square outperformed the tree diagram when referring to the students’ ability to quantify the subset relation that must be applied in Bayes’ rule. As hypothesized, in a second experiment with 143 students, the unit square was significantly more effective when the students’ performance in tasks based on Bayes’ rule was regarded. Our results could inform the debate referring to Bayesian reasoning since we found that the graphical transparency of nested sets could explain these visualizations’ effect.

## Introduction

As a part of Bayesian reasoning, the Bayes’ rule plays an important role in decision making under uncertainty. In many areas, such as medicine or law, critical decisions can depend on appropriately applying the Bayes’ rule, e.g., a medical diagnosis can depend on the probability of having a disease given a positive test result. Consider, for instance, a Bayesian reasoning situation like the following version of the medical diagnosis test situation without emphasizing a specific disease (Johnson and Tubau, 2015, p. 3):

“10% of women at age forty who participate in a study have a particular disease. 60% of women with the disease will have a positive reaction to a test. 20% of women without the disease will also test positive.”

In this situation, the probability that a woman who was selected at random and who received a positive test result actually has the disease can be calculated according to the Bayes’ rule. The resulting posterior probability P(H|D) where *H* is the hypothesis (having the disease) and *D* is the data (testing positive) is:

$$\begin{array}{c} P(H|D)=\frac{P(H)\cdot P(D|H)}{P(H)\cdot P(D|H)+P(\bar{H})\cdot P(D|\bar{H})}=\frac{10\%\cdot 60\%}{10\%\cdot 60\%+90\%\cdot 20\%}=25\% \end{array}$$

In general, people struggle when dealing with Bayesian reasoning situations (Kahneman et al., 1982). Particularly, most people, including physicians (Eddy, 1982), would expect a higher result for P(H|D) in medical diagnosis situations. Even professionals have trouble when they have to understand what a positive test result really means (e.g., Hoffrage and Gigerenzer, 1998; Ellis et al., 2014). Thus, the Bayes’ rule as a part of Bayesian reasoning is a topic that makes “clashes between intuition and probability” (Cosmides and Tooby, 1996, p. 2) apparent. Accordingly, in recent decades many scholars investigated ways of facilitating Bayesian reasoning (e.g., Mandel and Navarrete, 2015). Brase (2009, p. 369) stated that “certain basic mechanics of how to improve Bayesian reasoning have become clear over the past decade: use frequencies, use them in a nested subset framework and use pictures.” In the following, we regard these three aspects in a more detailed way and focus on the question of visualizing nested sets afterward.

The first aspect concerning “use frequencies” to facilitate Bayesian reasoning is a well-documented effect (e.g., Gigerenzer and Hoffrage, 1995; Cosmides and Tooby, 1996; Binder et al., 2015). Departing from an evolutionary point of view, Gigerenzer and Hoffrage (1995) and Cosmides and Tooby (1996) suggested the presentation of the statistical information in the format of natural frequencies instead of probabilities, since in our environment single-event probabilities are not observable and thus, during evolution, the human mind adapted to process natural frequencies rather than single-event probabilities. Using the information format of natural frequencies, the statistical information in the situation of medical diagnosis test mentioned above (Johnson and Tubau, 2015, p. 3) can be expressed as the following:

“10 out of 100 women at age forty who participate in a study have a particular disease. 6 out of 10 women with the disease will have a positive reaction to a test. 18 out of 90 women without the disease will also test positive.”

In this version, the required computation for the posterior probability P(H|D) is reduced to a simpler form of Bayes’ rule. Therefore, Johnson and Tubau (2015, p. 4) outline “the computational simplification afforded by natural frequencies” which is illustrated in following example when calculating the proportion of women having the disease among all women testing positive:

P(H|D)=66+18="6 out of 24"

The second aspect of using natural frequencies “in a nested subset framework” refers to a distinction of natural frequencies and normalized frequencies (e.g., Hoffrage et al., 2002) and to a debate about the nature of the facilitating effect of natural frequencies (e.g., Girotto and Gonzalez, 2001; Sloman et al., 2003; Barbey and Sloman, 2007; Gigerenzer and Hoffrage, 2007). In contrast to the evolutionary point of view, the cited research attributed the facilitating effect of natural frequencies to the fact that natural frequencies make the nested-set structure more salient and thus, it is easier to see how many events are in a subset and how the sets of events relate. The debate about related research was concluded with the statement that “there is wide consensus that natural frequency formats improve Bayesian performance by clarifying nested-set relations, which confers both representational and computational benefits” (Mandel and Navarrete, 2015, p. 2). Thus, one method to make nested-set structure transparent is to use natural frequencies instead of probabilities. This is, in some sense, a numerical way to make subset relations salient.

The third aspect concerning “use pictures” refers to the method of visualizing the statistical information. However, the facilitating effect of visualizations referring to Bayesian reasoning is ambiguous. First, visualizations that contain numerical information in the format of natural frequencies improved performance compared to text-only representations with natural frequencies (Wassner, 2004; Binder et al., 2015). But when the visualization contained the numerical information in the format of probabilities, there was only a small (Binder et al., 2015) or no effect (Sedlmeier and Gigerenzer, 2001, Study 2). Second, for visualizations that do not contain numerical information, the empirical evidence is mixed: Frequency grids improved performance in Garcia-Retamero and Hoffrage (2013) and, in an intervention study, in Sedlmeier and Gigerenzer (2001, Study 1); icon arrays had a beneficial effect in Brase (2009) but not in Sirota et al. (2014); Euler-diagrams (Brase, 2009) had no effect, and roulette-wheel diagrams (Brase, 2014) only a small effect.

These discrepancies raise the question which properties of visualizations are essential to facilitate Bayesian reasoning and under which conditions. Since it has been identified that bringing out the nested-set structure is important for the improvement in Bayesian reasoning tasks (Sloman et al., 2003), we focus in this article on the question of how visualizations make nested-set structure transparent. The numerical way to make nested-set structure transparent is the use of natural frequencies. Thus, our question is which graphical structure additionally, i.e., “beyond the effect of natural frequencies” (Garcia-Retamero and Hoffrage, 2013, p. 27), supports nested-sets transparency. For this purpose, we defined the criteria for the experimental conditions and the selection of specific visualizations for our study.

**FIGURE 1:**
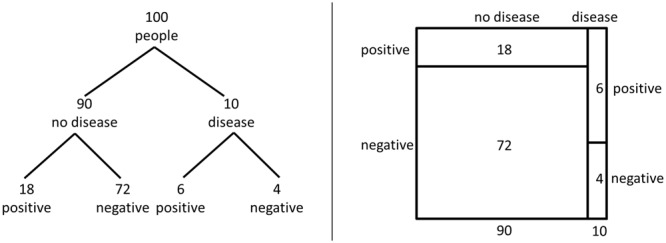
**Visualization of the statistical information for the medical diagnosis test problem in the version of Johnson and Tubau (2015, p. 3) with the tree diagram and the unit square, respectively**.

First, the visualization should include the statistical information in the form of natural frequencies. With this condition we kept the numbers constant across various visualizations of nested sets, thus we had a constant numerical transparency, but different graphical transparency of nested sets in the visualizations. Moreover, research results referring to the effectiveness of this feature of visualizations seem to be unambiguous (e.g., Wassner, 2004; Binder et al., 2015). According to this decision, we did not regard a test condition including text only, since research gave strong evidence that a text-only condition is not as effective as text with visualizations that show natural frequencies (ibid.). Further, we did not take into account icon arrays or frequency grids. Although research partly gave evidence about the effectiveness of those forms of visualization (e.g., icon arrays: Brase, 2009; frequency grids: Garcia-Retamero and Hoffrage, 2013), they were excluded in this study because they do not contain numerical information in the form of natural frequencies (except of hybrid versions; cf. Spiegelhalter et al., 2011). Second, there should be evidence that the selected visualizations for our study differ in their graphical effectiveness to make nested sets transparent. The second criteria resulted in the selection of the tree diagram and the unit square (**Figure 1**) due to the reasons outlined below.

Although the tree diagram is a common visualization of Bayesian reasoning situations or rather, repeated random events in statistics and statistics education (e.g., De Veaux et al., 2012) and many scholars use tree diagrams in their research papers to represent Bayesian reasoning situations (Gigerenzer and Hoffrage, 1995; Mandel, 2014; Navarrete et al., 2014), there are some hints in the literature suggesting that the tree diagram is not an ideal graphical nested-set representation. In an experiment based on probability and frequency instruction, the roulette-wheel diagram outperformed the tree diagram. This result was attributed to the clarity of the nested-set presentation (Yamagishi, 2003). Bea (1995) found in a training study with students of economics that the unit square with probabilities was more effective than the tree diagram with probabilities when student’s learning of conditional probabilities and the Bayes’ rule was considered. Moreover, double-tree diagrams with natural frequencies (Wassner, 2004) outperformed diagrams with only one natural frequency tree (Wassner, 2004). In Binder et al. (2015) the scores for 2 × 2-tables with natural frequencies were higher than the scores for the tree diagram with natural frequencies although this difference was not significant. Taking into account these hints, it is desirable to investigate more closely how tree diagrams reflect nested-set structure and to compare the effect of the tree diagram with other diagrams that reflect subset relations in another way.

The results of Bea (1995), Yamagishi (2003) and – more implicit – the results of Binder et al. (2015) could be interpreted as a result of a specific form of visualization. Thus, following Khan et al. (2015, p. 96) visualizations like the tree diagram represent a “Branch style” and emphasize relations of subsets in a logical way. By contrast, visualizations like the unit square, the roulette-wheel diagram, the Euler diagram and also the 2 × 2-table represent a “Nested style” and emphasize relations of subsets in a geometrical way. Based on a theoretical analysis of visualizations representing the “Nested style,” Oldford and Cherry (2006, p. 3) argued that especially the unit square “visually grounds probability and naturally incorporates the rules of probability within its construction” due to the area-proportionality of the unit square compared to the related statistical data. Whereas 2 × 2-tables can be understood to represent the “Nested style” according to Khan et al. (2015), they do not, in contrast to the unit square, include an area-proportionality. For this reason we hypothesize that a unit square represents in some sense an ideal way of visualizing nested sets.

According to the reasons for selecting specific visualizations for Bayesian situations, we investigate in this article how the nested-set structure is made transparent graphically by the tree diagram and the unit square based on natural frequencies. We first study the extent to which both visualizations make subset relations transparent and if this impacts the ability to quantify subset relations (Experiment 1). In a second step, we investigate the influence of both visualizations when using subset relations for solving Bayesian reasoning tasks (Experiment 2).

### Visualizing Nested Sets

Although the unit square and the tree diagram bear the same numerical information, they have quite different structures. The unit square is a statistical graph (Tufte, 2013) which means in the unit square, the sizes of the partitioned areas are proportional to the sizes of the represented data. Therefore, the unit square represents the proportion of subsets in a numerical and geometrical sense. In contrast, the tree diagram is not a statistical graph because the data is only represented by numbers. Consequently, the tree diagram represents the proportion of subsets only in a numerical sense. Even though the graphical representation of the size of the nodes could potentially be used to convey graphical information, this is rarely done in the literature (and even if one wanted to do this, it is not clear whether the radiuses of the nodes or their areas should be proportional to the numbers).

**FIGURE 2:**
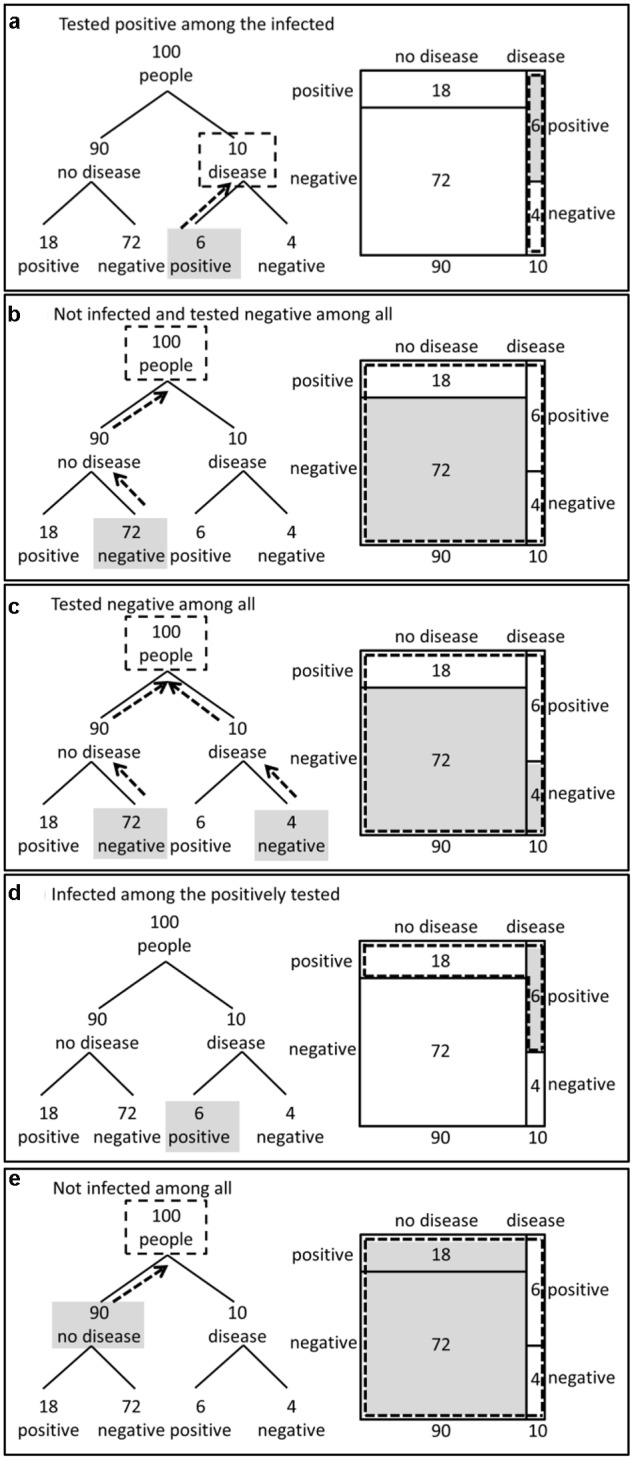
**Highlighting of subset relations in the tree diagram and the unit square**. The labels **(a–e)** indicate the structurally different subset relations in the 2 × 2-situation. Relation (d) involves the subset relation that has to be used when applying Bayes’ rule.

Although both the unit square and the tree diagram illustrate the nested-set structure of a Bayesian situation, they make nested-set structure transparent in different ways. Like Euler circles, the unit square shows the nested-set structure in the “Nested Style” (see introduction) by areas being included in other areas and therefore provides an image of sets being included in other sets. In **Figure 2**, we highlight different subset relations in the tree diagram and in the unit square. We arranged the subset relations in **Figure 2** in the same order as they were addressed later in our test-items in Experiment 1 (in contrast, there was no highlighting in the test-items). On the right side of **Figure 2**, we show how subset relations are graphically made transparent in the unit square: we highlight subsets by areas marked gray and sets by areas framed by dotted lines. In the unit square, subset relations can be grasped horizontally [e.g., subset relation (d) in **Figure 2**] as well as vertically [e.g., subset relation (a) in **Figure 2**]. The tree diagram, in contrast, represents the “Branch style” (see introduction) and visualizes the logical structure of subset relations by lines. The dotted arrows parallel to the branches in the tree diagrams (see **Figure 2**) highlight the logical relation between two sets when one set is the subset of another set. The tree implies a hierarchical structure and therefore only those subset relations that are in line with the hierarchy are graphically salient.

In the medical diagnosis situation, we have different sets and subsets (e.g., the set of infected people that are tested positive is a subset of all infected people). The subset relations shown in **Figure 2** are all possible subset relations that are structurally different. Any subset relation in a 2 × 2-situation has one of these five structures. Four of the subset-relations (i.e., a, b, c, and e, **Figure 2**) are in line with the hierarchy of the tree diagram. That means the sets including the subsets are on a higher level in the tree diagram than the subsets. Only the subset-relation “infected among the positively tested” (d, **Figure 2**) is not in line with the hierarchy of the tree diagram. Thus, for this subset relation, it is not possible to indicate the logical relation of being included by dotted arrows parallel to the branches. Moreover, there is no node in the tree diagram representing the set of all positively tested people that could be framed by a dotted line (d, **Figure 2**). Interestingly, it is exactly such a subset relation that is required when applying Bayes’ rule and calculating the posterior probability of being infected given that the test result is positive.

The differences in the properties of the two visualizations raise the question as to whether there is a difference between the unit square and the tree diagram when the perception of different subset relations is regarded. We presume that the unit square is more effective for subset relations that are not in line with the hierarchy of the tree diagram.

### Experiments

The aim of our research is to compare the effectiveness of the two visualizations in Bayesian reasoning situations, to understand which properties are essential and why differences occur. For this aim, we conducted two experiments. The method and procedure were similar for both experiments. In each experiment, we had two questionnaires, one showing unit squares to present the statistical information, the other showing tree diagrams. The tasks, the context stories and the numerical information were the same; only the visualization differed. Thus, we avoid any bias from the wording of the text or from question forms (c.f. Girotto and Gonzalez, 2001).

Since we decided to clearly concentrate our research on the effects of the visualization or rather of the properties of the visualizations, we give the statistical information only within the visualizations beside a short context story. Recent research results suggest that the performance of participants is not influenced by additionally giving all statistical information in a text (Binder et al., 2016). This illustrates that the choice not to provide additionally all statistical information as a text has no disadvantages. This way of presenting information in a Bayesian reasoning situation is slightly different from the majority of studies assessing the effect of visualization on Bayesian reasoning performance (e.g., Sedlmeier and Gigerenzer, 2001; Yamagishi, 2003; Brase, 2009; Micallef et al., 2012; Binder et al., 2015). These studies present statistical information in both texts and accompanying visual aids.

Further, in our research the relevant numbers have to be grasped from the visualizations. For this reason, someone could argue that effects measured referring to the performance in Bayesian reasoning tasks could quite simply be due to the effects of reading information. For this reason, we conducted a preliminary study to make sure that the unit square and the tree diagram were equally effective for extracting the relevant numbers; simple data are required for the numerator in the Bayes rule and sums over two summands are required for the denominator in the Bayes rule. For both reading of simple data and summarizing over extracted data, we can refer to a preliminary study with 77 undergraduates in which the unit square and the tree diagram were found to be equally effective (Böcherer-Linder et al., 2015). This was an important result because in our further steps of research we can exclude any bias from the effects of reading numerical information.

**FIGURE 3:**
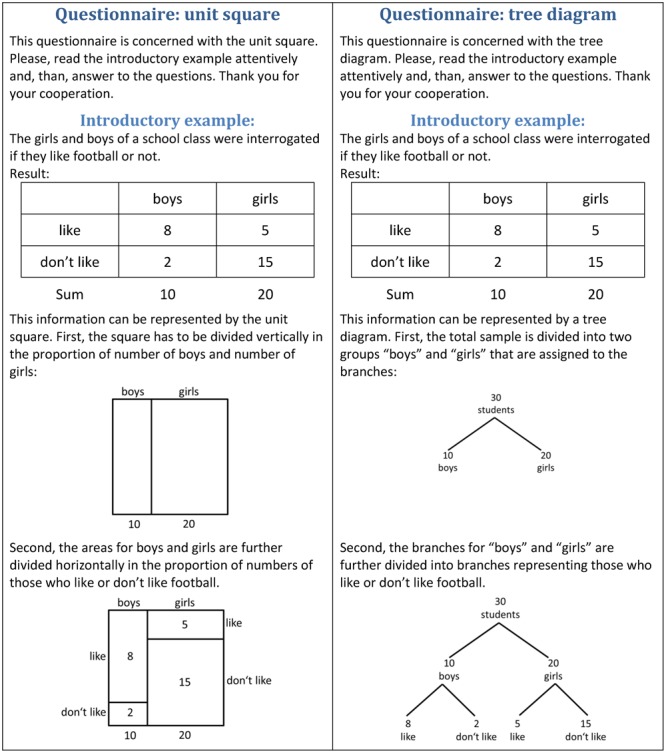
**Introductory examples for the unit square and for the tree diagram used in Experiments 1 and 2**.

To introduce the visualizations, we did not teach the participants how to read the visualizations, but we used the brief description shown in **Figure 3**. In both experiments, we used the same introductory example. Those participants who received the questionnaire with the unit square received the description of the unit square, those who received the questionnaire with the tree diagram received the description of the tree diagram. In the brief description, we first provided the statistical information in the form of a table that had similarities with the unit square (see **Figure 3**). However, in the preliminary study mentioned above where we used the same introductory example (Böcherer-Linder et al., 2015), participants’ ability to read out information from the visualizations did not differ. Thus, we concluded that this description was not an advantage in favor of the unit square.

Both experiments were carried out in accordance with the University Research Ethics Standards. Participation was voluntary and anonymity was guaranteed.

## Experiment 1

The first experiment was concerned with the graphical visualization of nested sets in the tree diagram and the unit square. More specifically, we had the following research question with the following hypotheses:

*Question 1*: Do unit squares and tree diagrams differ with respect to their ability to make subset relations transparent?

*Hypothesis 1a*: If the subset relation is in line with the hierarchy of the tree diagram, the unit square and the tree diagram are equally effective to make the subset relation transparent.

*Hypothesis 1b*: If the subset relation is not in line with the hierarchy of the tree diagram, the unit square is more effective to make the subset relation transparent.

### Method

#### Participants

The participants were 148 undergraduates (32 males, 115 females, 1 did not report the gender) at the University of Education Heidelberg (Germany). They were beyond their first semester of study and were enrolled in a mathematics education course. In this course, the two visualizations and the Bayes rule were not part of the curriculum. The participants were randomly assigned to the unit square (*N* = 74) and to the tree diagram (*N* = 74).

#### Materials and Procedure

To assess the perception of subset relations, we asked the students to calculate proportions and to indicate the result in fraction form. If proportions have to be calculated, someone must precisely determine the relation between the subset (numerator) and the set (denominator). Otherwise he or she is not able to calculate the following proportion:

$$\begin{array}{c} \mathrm{Proportion}=\frac{\#\mathrm{Subset}}{\#\mathrm{Set}} \end{array}$$

**FIGURE 4:**
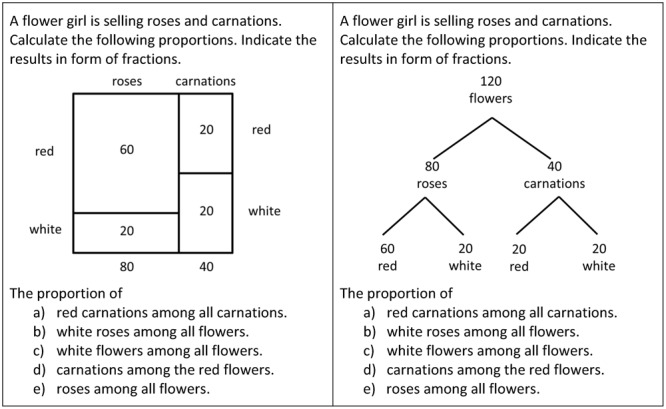
**The test-items of Experiment 1 for investigating students’ ability to quantify the five subset relations**.

Since we asked to indicate the result in fraction form, we could analyze if the correct subsets and correct sets have been grasped from the visualization. In **Figure 4** we provide our test items in both versions. The items (a) – (e) address exactly the subset relations (a) – (e) analyzed in Section 2, see **Figure 2**. Notice that the item (d) “carnations among the red flowers” asks for a subset relation that is not in line with the hierarchy of the tree diagram. The other items in contrast ask for subset relations that are in line with the hierarchy of the tree diagram. For each item, correct answers (i.e., correct numerator and correct denominator) were rated with 1, incorrect answers with 0. The participants worked individually.

### Results of Experiment 1

**FIGURE 5:**
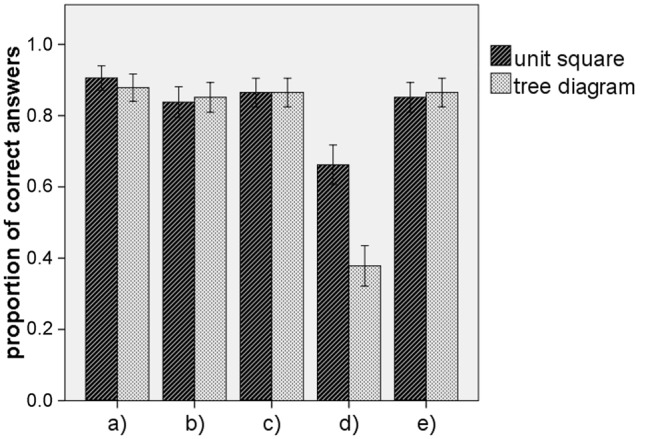
**Participants performance of quantifying the five subset relations in Experiment 1**. The five categories (a–e) at the *x*-axes correspond to those displayed and explained in **Figures 2** and **4**. The error bars indicate one standard error of the mean.

**Figure 5** illustrates the results of Experiment 1 for each of the items (a) – (e). To test the hypothesis, we first investigated a potential difference for all four items for which the subset relation was within the hierarchy of the tree diagram. For the accumulated score referring to these four items (Cronbach’s α = 0.739) a *t*-test yielded no significant difference between the tree diagram (*M* = 3.46, *SD* = 1.023) and the unit square (*M* = 3.46, *SD* = 1.036), *t*(146) = 0.000, *p* = 1.000. In addition, we tested each of the four sub-items a, b, c, and e individually. None of the items yielded a significant difference between the unit square and the tree diagram (see **Table 1**). Thus, there was no reason to reject our *Hypothesis 1a*. Interestingly the mean values of correct answers for the tree diagram were almost equal and the proportion of correct answers was very high for each of the four items addressing subset-relations that were in line with the hierarchy of the tree diagram (see **Table 1**; **Figure 5**).

**Table 1:** Results of *t*-tests for each of the items concerning the subset relations in Experiment 1.

Perceiving subset relations									
**Items**	**a**		**b**		**c**		**d**		**e**	
**Diagram**	**Square**	**Tree**	**Square**	**Tree**	**Square**	**Tree**	**Square**	**Tree**	**Square**	**Tree**
*M*	0.91	0.88	0.84	0.85	0.86	0.86	0.66	0.38	0.85	0.86
*SD*	0.295	0.329	0.371	0.358	0.344	0.344	0.476	0.488	0.358	0.344
*t*	0.526		-0.225		0.000		3.579		-0.234	
*df*	146		146		146		146		146	
*p*	0.599		0.822		1.000		<0.001		0.815	

The item (d) addressed a subset relation that was not in line with the hierarchy of the tree diagram. Here, the unit square (*M* = 0.66, *SD* = 0.476) was more effective than the tree diagram (*M* = 0.38, *SD* = 0.488), *t*(146) = 3.579, *p* < 0.001, with an effect size of *d* = 0.58. Thus, our *Hypothesis 1b* was confirmed.

Note, even if we tested the results by referring to each of the five items as representing different subset-relations, using the Bonferroni adjustment, the difference between the effectiveness of the tree diagram and the unit square concerning item (d) is still significant, *p*^∗^ = 5; *p* < 0.01.

## Experiment 2

The second experiment was concerned with the impact of graphical visualization of nested sets on performance in Bayesian reasoning tasks. More specifically, we had the following research question with the following hypothesis:

*Question 2*: Do unit squares and tree diagrams differ with respect to performance in Bayesian reasoning tasks?

*Hypothesis 2*: The unit square is more effective than the tree diagram with respect to performance in Bayesian reaoning tasks.

Note, that the *Hypothesis 2* of Experiment 2 is a logical consequence of the result of Experiment 1, because the subset relation that is required for the Bayes’ rule is not in the line with the hierarchy of the tree diagram.

### Method

#### Participants

The participants were 143 undergraduates (125 males, 18 females) at the Technical University of Munich (Germany). They were in the fourth semester of their study and were enrolled in an Electrical Engineering course. In this course, the two visualizations and the Bayes rule were not part of the curriculum. The participants were randomly assigned to the unit square (*N* = 74) and to the tree diagram (*N* = 69).

#### Materials and Procedure

**FIGURE 6:**
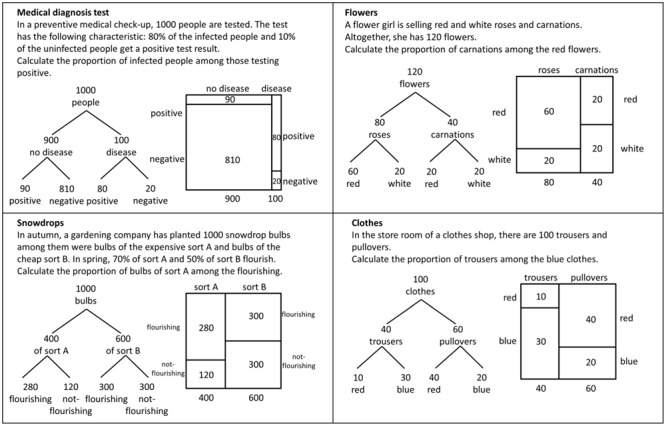
**The test-items of Experiment 2 for investigating students’ performance when solving Bayesian reasoning tasks.** The original test-items showed either the tree diagram or the unit square.

We had four test items concerning Bayes’ rule (see **Figure 6**). We did not focus on the interpretation of probability but on the computations for Bayes’ rule and therefore asked to calculate proportions. The answer was rated with 1 for the correct value of the proportion, no matter if the value was indicated in the form of fraction, percentage or decimal number. The answer was rated with 0 when the value for the proportion was incorrect.

For the presentation of the statistical information in the items, we decided to describe the Bayesian situations in such a way that the problems could only be solved by reading the information from the visualizations (see the beginning of the experiment section). Therefore we did not provide natural frequencies in the text (except the total sample size). In the items “medical diagnosis test” and “snowdrops” (see **Figure 6**), we only gave some information characteristic of a Bayesian situation (like sensitivity or specificity of a test) in a normalized form as percentages. In two further items (“flowers” and “clothes”) we even reduced the statistical information in the text and only indicated the size of the sample (see **Figure 6**). The tasks for the unit-square group and for the tree-diagram group were identical, only the visualizations presenting the statistical information differed between the two groups.

When working on the questionnaire, participants were sitting close to each other. Thus, to avoid that participants assigned to the unit square and participants assigned to the tree diagram would be influenced by each other, we had different orders in the items: The order of the tasks for the unit square was “flowers,” “medical diagnosis,” “clothes,” and “snowdrops.” The order of the tasks for the tree diagram was “clothes,” “medical diagnosis,” “flowers,” and “snowdrops.” We can assume that no bias was introduced by the slightly different order, because in a pilot study (Böcherer-Linder et al., 2015) where we used four questionnaires A (tree diagram), B (unit square), C (tree diagram), and D (unit square) with the same order of items in A and B and the same order of items in C and D but different to A and B, we found that the performance was not influenced by the order of the items.

The procedure of Experiment 2 was the same as in Experiment 1 and we used the same introductory examples as in Experiment 1 as front pages of the questionnaires.

### Results of Experiment 2

**FIGURE 7:**
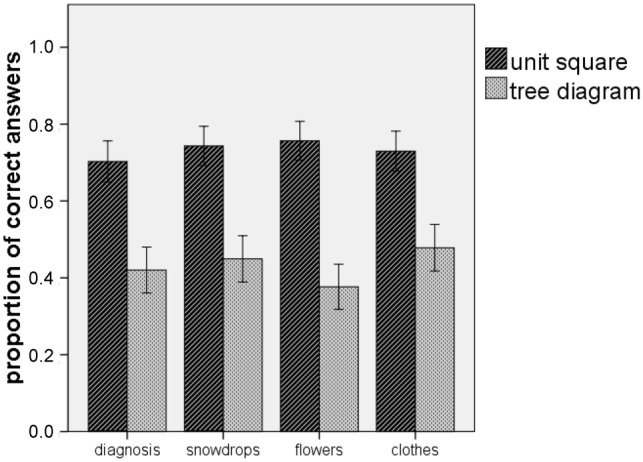
**Participants performance when solving Bayesian reasoning tasks in Experiment 2.** The error bars indicate one standard error of the mean.

**Figure 7** illustrates the results of Experiment 2. For each of the four items, the unit square was significantly more effective with medium to large effects (see **Table 2**). Moreover, for the accumulated scores of the four items (α = 0.807), the unit square (*M* = 2.93, *SD* = 1.417) was more effective than the tree diagram (*M* = 1.72, *SD* = 1.494), *t*(141) = 4.961, *p* < 0.001, with a large effect size of *d* = 0.84. We can conclude that, whether the information in the text was reduced or not, the unit square was more effective than the tree diagram when the performance in Bayesian reasoning tasks was considered. Thus, the second hypothesis was confirmed.

**Table 2:** Results of *t*-tests for each of the items concerning performance in Bayesian reasoning tasks in Experiment 2.

Solving Bayesian reasoning tasks									
**Item**	**Diagnosis**		**Snowdrops**		**Flowers**		**Clothes**	
**Diagram**	**Square**	**Tree**	**Square**	**Tree**	**Square**	**Tree**	**Square**	**Tree**
*M*	0.70	0.42	0.74	0.45	0.76	0.38	0.73	0.48
*SD*	0.460	0.497	0.440	0.501	0.432	0.488	0.447	0.503
*t*	3.518		3.718		4.915		3.150	
*df*	137.999		135.607		136.011		136.212	
*p*	0.001		<0.001		<0.001		0.002	
*Cohens’ d*	0.59		0.62		0.83		0.53	

For both visualizations, additional analyses revealed no statistical differences between the scores for the items with different presentations of the statistical information in the text. This is in line with a study of Binder et al. (2016) where the additional presentation of all statistical information in the text did not influence the performance of the participants.

## Results and Discussion

There is some evidence that visual aids can increase performance in Bayesian reasoning tasks even when the statistical information is given in terms of natural frequencies (cf. Garcia-Retamero and Hoffrage, 2013). However, it is still an important question, why a specific visualization could facilitate understanding in Bayesian reasoning situations (e.g., Sirota et al., 2014). For this reason, we focused particularly on the question, which graphical properties of visualizations yield an effect on solving tasks in Bayesian reasoning situations in addition to the property of displaying the statistical information in form of natural frequencies. For visualizations that contain natural frequencies the beneficial effect was proven compared to text-only conditions (e.g., Wassner, 2004; Binder et al., 2015). Since Sloman et al. (2003) stated that the extent of making the nested-set structure of a Bayesian reasoning situation transparent strongly impacts the performance of solving tasks in these situations, we particularly focused on the visualizations’ graphical property to make nested-set structure transparent.

We showed theoretically that the tree diagram and the unit square make the nested-set structure in Bayesian reasoning situations transparent in different ways. The tree diagram representing a “Branch style” (Khan et al., 2015) does not visualize the subset-relation that is necessary for applying Bayes’ rule whereas the unit square representing a “Nested style” (ibid.) visualizes this subset-relation by neighboring areas. According to this theoretical consideration, Experiment 1 yielded a main result of our research: it is important to differentiate between different subset relations when regarding the graphical transparency of nested sets in visualizations. Whereas subset relations that are in line with the hierarchy of the tree diagram are graphically salient and therefore produced high performance, subset relations that are not in line with the hierarchy of the tree diagram were not graphically salient (see **Figure 2**) and resulted in a much lower performance. Thus, there is not simply graphical transparency of nested-set structure in visualizations, but more precisely graphical transparency of certain subset relations. By contrast, in the unit square every subset relation is visualized by neighboring areas. Accordingly, a further main result of Experiment 1 was that the unit square outperformed the tree diagram when participants had to quantify the subset relation that is required for the Bayes’ rule. Moreover, the results offer a possible explanation for this effect: If the cause of the benefit of the unit square compared to the tree diagram had been due to the redundant geometrical and numerical magnitude representation, we would have expected the supremacy of the unit square for all the different subset relations in Experiment 1. However, since the unit square is only predominant for subset relations that are not in line with the hierarchy of the tree diagram, we attribute this effect to the graphical transparency of the relevant subset relation and not to the area-proportionality. Therefore, we can argue that the measured effect is not due to a possible “frequentist reading” (Moro et al., 2011, p. 849) of the unit square.

As a consequence of the graphical transparency of the relevant subset relation, the unit square outperformed the tree diagram in all of the four Bayesian reasoning situations in Experiment 2. We interpret this result as based on the graphical properties since both visualizations include the statistical information in the form of natural frequencies. Therefore, the unit square makes the nested-set structure of the problem transparent to a greater extent (cf. Sloman et al., 2003; Barbey and Sloman, 2007).

Our findings are in accordance with other research findings and could serve as an interpretation of existing research findings. For example, Yamagishi (2003, p. 103) found that the roulette-wheel diagram was more effective than the tree diagram in both frequency and probability conditions and attributed the “roulette-wheel diagram’s supremacy over the frequency tree” to its graphical nature that “symbolizes the relevant nested-sets relations” (p. 105). This finding could be explained by our results showing that the relevant subset relation in the tree diagram is not graphically transparent. Wassner (2004) reported that double-tree diagrams with natural frequencies were more effective than tree diagrams with natural frequencies as visualizations of a medical diagnosis situation. Our results suggest that the double-tree diagram makes the relevant subset relation more transparent compared to the tree diagram, because the set of all positively tested people is represented by one node in the double-tree which is missing in the tree diagram (see **Figure 2**). Moreover, frequency grids containing natural frequencies in a legend were equally effective when compared to double-trees containing the same numerical information in Khan et al. (2015). Therefore, we would expect that frequency grids containing natural frequencies in a legend are more effective than tree diagrams with natural frequencies, as it is the case for double-trees. In consequence, given the graphical similarities that the unit square shares with the frequency grid, we would expect that the unit square with natural frequencies is equally effective in comparison to the frequency grid with a legend. The results of Sedlmeier and Gigerenzer (2001, Study 1) implied no difference between the frequency grid and the tree diagram. However, this might be an effect of the training or the small sample (seven participants for the frequency grid and five participants for the tree diagram, in the third session, Sedlmeier and Gigerenzer, 2001, p. 388).

Given our results, it might be interesting for future research to compare unit squares and 2 × 2-tables for two reasons. First, unit squares and 2 × 2-tables are closely related; the only difference is that unit squares additionally mirror statistical information geometrically. Second, the results of Binder et al. (2015, p.6) suggest an advantage of the 2 × 2-table compared to the tree diagram, although no statistical difference between 2 × 2-tables and tree diagrams was reported (see Introduction). We hypothesize that the area-proportionality of the unit square could have an additional beneficial effect compared to the advantage of the 2 × 2-table. It could be also an interesting question to compare the unit square (with natural frequencies) and frequency grids that Khan et al. (2015, p. 96) called “Frequency style” and that proved to be effective for understanding Bayesian reasoning situations (Garcia-Retamero and Hoffrage, 2013; Khan et al., 2015). As mentioned above, we hypothesize that both forms of visualization show the same effectiveness.

For the estimation of the beneficial value of visualizations it is also important to take into account the different aims visualizations can have in the context of applied uses. For example, every data-proportional display, and thus the unit square and also icon arrays or frequency grids, are limited in terms of displaying extreme values in statistical information that could occur when the base rate is extremely low. In this case, visualizations representing the Branch style (e.g., a tree diagram) and containing natural frequencies have the advantage of representing the statistical data only numerically. A further aim of visualization is to facilitate learning and, for this purpose, to build up conceptual knowledge (Rittle-Johnson and Schneider, 2015). For this aim, however, it is not necessarily needed to draw the unit square exactly true to scale. The understanding of the structure of the data can be supported as well by a rough drawing of a unit square which can be easily made by hand. Thus, the limitation of displaying extreme values is not a limitation of developing conceptual knowledge about Bayesian situations. Nevertheless, further research is needed to know more precisely in which situations and for which people the unit square is particularly helpful. For example, due to the geometrical representation of numerical information, we assume that the unit square could be very helpful in situations when the understanding of the structure of the data is demanded. The results of Bea (1995), who used the unit square with probabilities in a training study provides evidence for this assumption. Further, the unit square offers the possibility to be realized as an interactive visualization which turned out to be an effective tool in Tsai et al. (2011) and could be advantageous for the understanding of parameter dependency in Bayesian reasoning situations. However, it is an open question if participants’ numeracy or spatial abilities have an influence on the effect of the unit square.

## Conclusion

Our results show that it is important to analyze the properties of visualizations that potentially could facilitate Bayesian reasoning (cf. also Brase, 2009; Micallef et al., 2012). In our study we focused on the property to visualize the nested-set structure of a Bayesian reasoning situation. Our results show further that the graphical visualization of nested sets impacts performance in Bayesian reasoning tasks. Finally, we showed that the unit square representing a “Nested style” is an effective visualization of Bayesian reasoning situations and can be used as a flexible display for risk communication as well as for mathematics education.

## Author Contributions

All authors listed, have made substantial, direct and intellectual contribution to the work, and approved it for publication.

## Conflict of Interest Statement

The authors declare that the research was conducted in the absence of any commercial or financial relationships that could be construed as a potential conflict of interest.
